# P53 maintains gallid alpha herpesvirus 1 replication by direct regulation of nucleotide metabolism and ATP synthesis through its target genes

**DOI:** 10.3389/fmicb.2022.1044141

**Published:** 2022-11-24

**Authors:** Li Xu, Zhijie Chen, Yu Zhang, Lu Cui, Zheyi Liu, Xuefeng Li, Shengwang Liu, Hai Li

**Affiliations:** ^1^State Key Laboratory of Veterinary Biotechnology, National Poultry Laboratory Animal Resource Center, Harbin Veterinary Research Institute, Chinese Academy of Agricultural Sciences, Harbin, China; ^2^Department of Pathogenic Microbiology and Immunology, School of Basic Medical Sciences, Xi’an Jiaotong University, Xi’an, China

**Keywords:** alphaherpesviruses, P53, PFT-α, virus-host interactions, virus replication

## Abstract

P53, a well-known tumor suppressor, has been confirmed to regulate the infection of various viruses, including chicken viruses. Our previous study observed antiviral effect of p53 inhibitor Pifithrin-α (PFT-α) on the infection of avian infectious laryngotracheitis virus (ILTV), one of the major avian viruses economically significant to the poultry industry globally. However, the potential link between this antiviral effect of PFT-α and p53 remains unclear. Using chicken LMH cell line which is permissive for ILTV infection as model, we explore the effects of p53 on ILTV replication and its underlying molecular mechanism based on genome-wide transcriptome analysis of genes with p53 binding sites. The putative p53 target genes were validated by ChIP-qPCR and RT-qPCR. Results demonstrated that, consistent with the effects of PFT-α on ILTV replication we previously reported, knockdown of p53 repressed viral gene transcription and the genome replication of ILTV effectively. The production of infectious virions was also suppressed significantly by p53 knockdown. Further bioinformatic analysis of genes with p53 binding sites revealed extensive repression of these putative p53 target genes enriched in the metabolic processes, especially nucleotide metabolism and ATP synthesis, upon p53 repression by PFT-α in ILTV infected LMH cells. Among these genes, eighteen were involved in nucleotide metabolism and ATP synthesis. Then eight of the 18 genes were selected randomly for validations, all of which were successfully identified as p53 target genes. Our findings shed light on the mechanisms through which p53 controls ILTV infection, meanwhile expand our knowledge of chicken p53 target genes.

## Introduction

Avian infectious laryngotracheitis virus (ILTV), also known as gallid alphaherpesvirus 1 (GaHV-1), is a member of the family *herpesviridae* and the subfamily *alphaherpesvirinae*. Despite widespread immunization, ILTV infection still result in avian infectious laryngotracheitis (AILT), which leads to significant financial losses for the global chicken industry (Ou and Giambrone, 2012). The major method for preventing and controlling AILT at current stage is vaccination with ILTV-attenuated vaccines (Rodríguez-Avila et al., 2007; García and Zavala, 2019; Thilakarathne et al., 2019). However, with successive passage in the host, the attenuated viruses may regain virulence through the recombination of viral genome between different ILTV strains, including vaccine strains, which would cause new epidemic of AILT (Lee et al., 2012; Fakhri et al., 2020). Additionally, similar to human alphaherpesviruses, ILTV is hard to be eliminated from the host and there is presently no viable therapeutic cure due to the formation of latent infection in the trigeminal ganglia following acute infection (Thilakarathne et al., 2019). As a result, ILTV may reactivate from a latent infection once host immunity is impaired (Coppo et al., 2013; Adler et al., 2017). Thus, it is anticipated that research into ILTV and host interaction mechanisms will help to develop therapeutics independent of the host immune response and serve as a complement to present AILT control.

P53 is one of the most famous tumor suppressors, as proofed by its dysfunction in most malignancies (Menendez et al., 2009). P53 has been shown to control the infections of several viruses, including Epstein-Barr Virus (Kraus et al., 2020), Japanese encephalitis virus (Wang et al., 2020), hepatitis B virus (Song et al., 2021), and Porcine Epidemic Diarrhea Virus (Su et al., 2021). In chickens, the important role of p53 in ALV-J infection has been evidenced by the mutations associated with abnormal expression of chicken p53 in ALV-J-associated myelocytomas (Yue et al., 2015) and the promoted ALV-J replication upon p53 knockout (Zhang et al., 2021). The same phenomenon is observed in chickens infected with infectious bursal disease virus. The overexpression of chp53 inhibited IBDV replication, whereas chicken p53 inhibition led to the opposite effect (Ouyang et al., 2017). Although p53 was initially observed only through the anti-tumor effect of p53 to regulate metabolic genes, it is becoming increasingly clear that p53’s role in regulating metabolism goes far beyond its role in tumor suppression (Maddocks and Vousden, 2011). Nucleotide biosynthesis is essential for supporting DNA replication and RNA synthesis in cancer cells that proliferate quickly. P53 inhibits purine synthesis and cell proliferation by inhibiting GMP synthase (GMPS), a key enzyme in the purine synthesis pathway (Holzer et al., 2017). P53 can also suppress ribonucleotide reductase through inhibiting mTORC1 to restrain the key steps in nucleotide synthesis (He et al., 2017). It is worth noting that p53 controls the effector process by activating multiple target genes (Kastenhuber and Lowe, 2017). In our previous research, we conducted comprehensive characterization of the genome-wide chromatin occupancy of chicken p53 and identified 754 direct target genes of chicken p53 (Chen et al., 2022), which may advance our comprehension of the p53 transcriptional regulatory mechanisms in chickens. Given p53’s significant function, understanding the processes driving p53-mediated biological consequences is crucial for the development of p53-based therapeutics.

PFT-α has been shown to inhibit the transcriptional activity of p53 in both human and chicken cell lines (Li et al., 2018; Garbern et al., 2020; Xu et al., 2022). Our earlier research revealed that p53 repression leaded to paracrine-regulated apoptosis of bystander cells during ILTV infection together with abnormal metabolism in these cells (Li et al., 2018). This is in line with our recent study that the p53 inhibitor PFT-α inhibited nucleotide metabolism and ATP synthesis in host cells infected with ILTV (Xu et al., 2022). Here, we examined the impacts of host p53 knockdown on each stage of ILTV replication and explored its underlying mechanisms by transcriptome analysis in combination with chromatin immunoprecipitation (ChIP) assays using chicken LMH cell line as the model (Mahmoudian et al., 2012; Bendezu et al., 2019; Qiao et al., 2020).

## Materials and methods

### Viral strain and cells

The ILTV strain ILTV-LJS09 propagated in leghorn male hepatoma (LMH) cells was described in previous studies (Kong et al., 2013; Xu et al., 2022). Dulbecco’s modified Eagle’s medium (DMEM) was used to sustain LMH cells (ATCC CRL-2117), and 10% fetal bovine serum (FBS), 100 units/mL penicillin, 100 g/mL streptomycin, and 2 mM L-glutamine were supplemented. Cell cultures were maintained at 37°C with 5% CO_2_. The final concentration of PFT-α (Sigma-Aldrich Corporation) utilized was 20 μM after being dissolved in DMSO (Sigma-Aldrich Corporation). No more than 0.1% of DMSO was added in cell culture in all groups, including the control group.

### RNA interference and transfection

Three short-interfering RNAs (siRNAs) that precisely targeting chicken *TP53* mRNA sequence (NM_205264; sip53-1, 5′-AGG AGG AGA ACU UCC GCA A-3′; sip53-2, 5′-GCG UGG CUA AGC GAG CCA U-3′; sip53-3, 5′-GCU GCU UCG AGG UGC GCG U-3′) were synthesized. A siRNA with no target site in any chicken mRNA (sicontrol, 5′-GCA CUU GAU ACA CGU GUA A-3′) was used as control. All siRNAs were ordered from Sigma-Aldrich Corporation. siRNA transfection was performed with a Lipofectamine^®^ 2000 Transfection Reagent (Invitrogen, Carlsbad, CA, USA).

### Plasmids and transfection

The recombinant plasmid Flag-chp53 was provided by Dr. Zhiyong Ma and Dr. Yafeng Qiu (Shanghai Veterinary Research Institute, CAAS). The transfection was carried out as described in our previous study (Chen et al., 2022), using Turbofect transfection reagent (Thermo Fisher Scientific, Rockford, IL).

### RNA extraction and real-time quantitative PCR

Total RNA was extracted from LMH cells using RNAiso Plus (Takara Biotechnology, Dalian, China) at the given time points after infection, in accordance with the manufacturer’s procedure. Cells were infected with ILTV at a multiplicity of infection (MOI) of 1. A microvolume spectrophotometer was used to assess the concentration and quality of total RNA (Implen GmbH, Munich, Germany). The program Oligo 7 was used to create the primer sequences (version 7.6.0, Molecular Biology Insights, Inc., Colorado Springs, CO). The sequences of primers are presented in Supplementary Table 1. A One Step TB Green^®^ PrimeScript™ RT-PCR Kit II was used to for real-time quantitative PCR (RT-qPCR) (Takara Biotechnology, Dalian, China). Each sample was measured three times. By 2^–ΔΔCt^ approach, the relative quantification of target gene expression was computed using β*-actin* as the endogenous control, and the data are shown as the log_2_ fold change. With three technical duplicates for every reaction, at least three separate experiments were carried out.

### Viral quantitation

Plaque tests and ILTV-specific RT-qPCR assays were used to measure the amounts of viral replication, as previously described (Qiao et al., 2020). AxyPrep Body Fluid Viral DNA/RNA Miniprep Kit was used for genomic DNA extraction (Axygen, Union City, CA, USA). Using Luna Universal qPCR Master Mix (NEB, Ipswich, MA, USA), absolute RT-qPCR was carried out in accordance with the manufacturer’s instructions. pMD18-T plasmid containing *gC* genes of ILTV was used as standards. Three or more independent experiments were performed for each experiment.

### Western blot analysis

Western blot was performed strictly according to previously described procedures (Chen et al., 2022). Briefly, cells were washed with ice-cold PBS and soluble proteins were extracted with RIPA Lysis Buffer (Strong) (Beyotime Biotech, Shanghai, China) according to the manufacturer’s protocol. The protein concentration of each sample was determined by a BCA Kit (Beyotime Biotech, Shanghai, China). An equal amount of protein was separated by SDS-PAGE and transferred onto a nitrocellulose membrane (Millipore, Billerica, MA). The membrane was then blocked with 5% non-fat milk for 2 h at room temperature and incubated with primary antibodies overnight at 4°C. Antibodies against P53 (Santa Cruz Biotechnology, Santa Cruz, USA) and β-actin (Proteintech, Wuhan, China) were used. Signals were visualized using infrared imaging systems (Odyssey CLX, LiCor Biosciences, Lincoln, NE).

### RNA sequencing

The p53 inhibitor PFT-α (Sigma-Aldrich Corporation) were used at 20 μM before ILTV infection. Control groups were treated with DMSO at the same volumes. After the administration of PFT-α for 12 h, Cells were infected with ILTV at a multiplicity of infection (MOI) of 1 and cells were collected at 12 h post infection (hpi). Annoroad Gene Technology Co., Ltd. used RNA deep sequencing to undertake genome-wide gene expression profiling of LMH cells (Beijing, China). The Illumina platform (Illumina, Inc., San Diego, CA, USA) was used to build the library in accordance with the manufacturer’s guidelines. With the use of an Illumina HiSeq 2500 device, the samples were sequenced. There were four biological repetitions.

### High-throughput data analysis

The web-based software Galaxy was used to analyze the results of RNA sequencing (Blankenberg et al., 2010). The DESeq R package was used to analyze DEGs and the read-count data obtained by analyzing gene expression levels were standardized. The hypothetical probability (*p*-value) was calculated according to the negative binomial distribution model. The screening criteria for DEGs were *p*-value < 0.05 and | log_2_ (fold change) | > 1. KEGG pathway enrichment analysis was used to determine the primary functions of DEGs using DAVID (gene-enrichment analysis using EASE Score, a modified Fisher exact *p*-value, as the threshold) (Huang et al., 2009). Additionally, a corrected *p*-value < 0.05 was the threshold for a statistically significant correlation. RNA sequencing raw data were uploaded to the National Center for Biotechnology Information database (GSE193188).

### Chromatin immunoprecipitation assays

ChIP assays were performed according to previous description (Chen et al., 2022). For each sample, 5 × 10^6^ cells were collected. A total of 5 μg of anti-flag (GenScript, Piscataway, NJ, USA) or isotype control IgG2b antibody (Cell Signaling Technology, Danvers, Massachusetts, USA) was used in each sample. Pull-down was performed using Protein A/G PLUS-agarose beads (Santa Cruz Biotechnology, Santa Cruz, USA). A QIAquick PCR Purification Kit was used to purify the immunoprecipitated DNA (QIAGEN, Valencia, CA). Using Luna Universal qPCR Master Mix (NEB, Ipswich, MA, USA), ChIP followed by Quantitative PCR (ChIP-qPCR) was carried out according to previous description (Chen et al., 2022). The sequences of the primers are shown in Supplementary Table 2. Three biological repetitions were performed.

### Statistical analysis

All statistical analyses were performed using the SPSS software suite (SPSS for Windows version13.0, SPSS Inc., Chicago, IL, USA). The mean ± standard deviation (SD) was used to present most data. The significance of differences between two groups was determined using two-tailed Student’s *t*-test.

## Results

### P53 is a host determinant of viral gene transcription and subsequent viral replication of infectious laryngotracheitis virus

**FIGURE 1:**
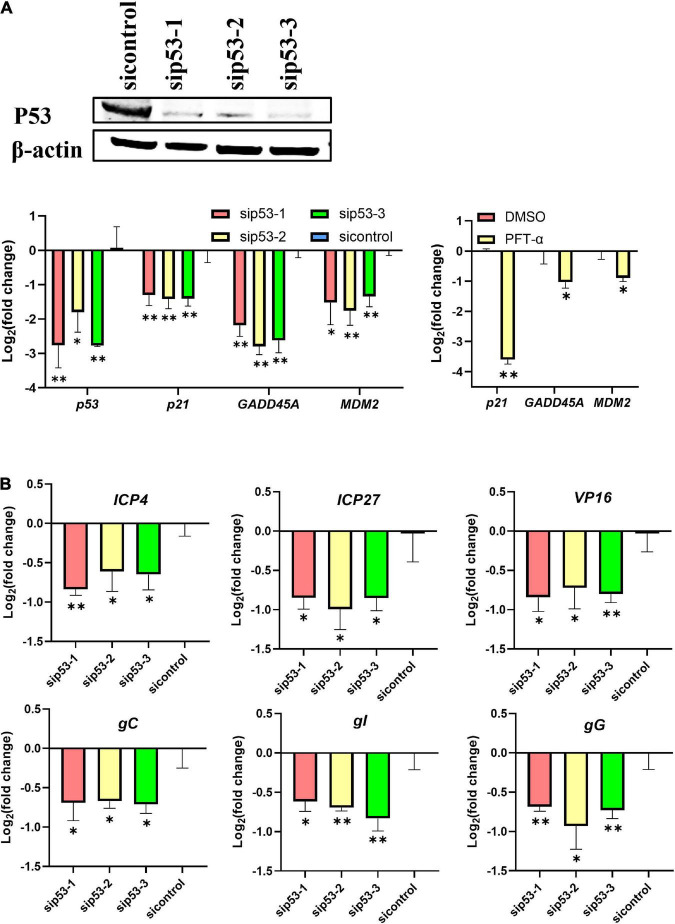
P53 is a host determinant of ILTV gene transcription in LMH cells. LMH cells were transiently transfected with p53 siRNA (sip53) or negative control siRNA (sicontrol) for 24 h. **(A)** The knockdown efficiencies of p53 siRNA were analyzed by immunoblotting and RT-qPCR and the effect of PFT-α on p53 target genes were analyzed by RT-qPCR. **(B)** The transcription levels of six ILTV genes covering all stages of ILTV transcription, namely *ICP4*, *ICP27*, *VP16*, *gC, gI*, and *gG*, in LMH cells were quantitated 6 h after ILTV infection (MOI = 1) using RT-qPCR. The results are presented as the mean ± SD, *n* = 3. *p < 0.05 and **p < 0.01 indicated the levels of significance.

**FIGURE 2:**
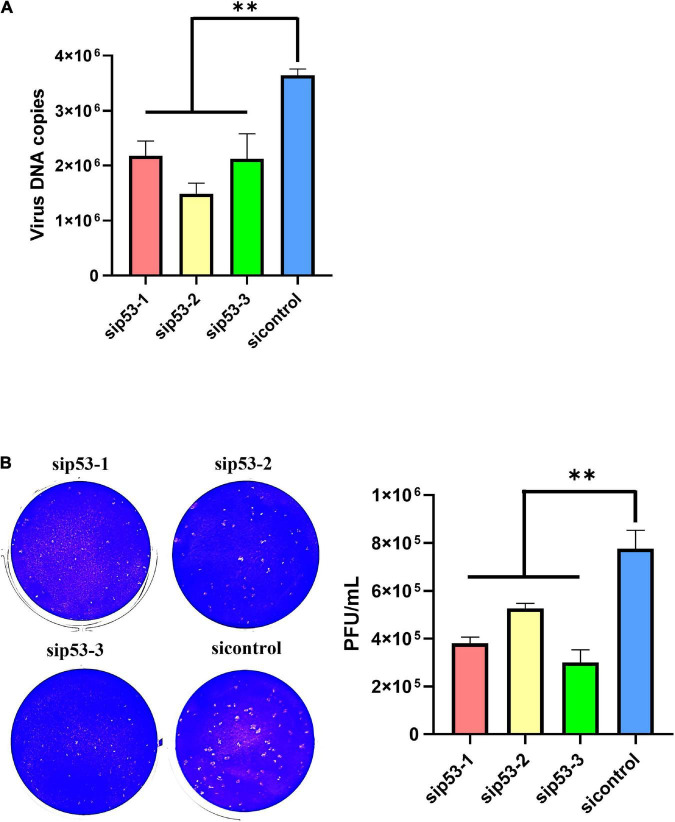
P53 is a host determinant of ILTV replication in LMH cells. **(A)** At 12 hpi, viral genome copy numbers were determined by ILTV-specific RT-qPCR assays. **(B)** The number of infectious virions was quantified by plaque assays at 3 dpi. The results are presented as the mean ± SD, *n* = 3. **p < 0.01 indicates the levels of significance.

P53 was knocked down in LMH cells using siRNAs before ILTV infection to clarify the impact of p53 on ILTV replication. Western blotting and RT-qPCR was performed to assess the knockdown efficacy (Figure 1A). *P21*, *MDM2*, and *GADD45A* have been identified as p53 target genes experimentally in chicken (Deng et al., 2010; Yang et al., 2013). Their transcription were detected by RT-qPCR to determine p53 transcriptional activity. β*-actin* was served as inner control. Upon p53 knockdown, the transcription of these p53 target genes were all repressed, suggesting successful inhibition of p53 transcriptional activity by knockdown (Figure 1A). The inhibition of p53 transcriptional activity by PFT-α was evidenced by RT-qPCR, which revealed significant downregulation of three well-known p53 target genes, *p21*, *GADD45A*, and *MDM2*, by the administration of PFT-α (Figure 1A). In LMH cells 6 h post infection (hpi) with ILTV, p53 knockdown significantly reduced the transcription of the immediate-early gene (IEG) *ICP4*, the early genes (EG) *ICP27*, *VP16*, and *gC*, the early/late gene (E/LG) *gI*, and the late gene (LG) *gG* (Mahmoudian et al., 2012), as determined by RT-qPCR, suggesting that p53 plays a regulatory role in the ILTV gene transcription (Figure 1B). Further, p53 knockdown significantly reduced subsequent viral genome replication and virion production, as determined by viral DNA detection using ILTV-specific RT-qPCR (Figure 2A) in LMH cells at 12 hpi and plaque assays in LMH cells at 3 dpi (Figure 2B), respectively. The titer of infectious virions in LMH cells was 7.77 × 10^5^ PFU/mL at 3 dpi but was 3.8 × 10^5^ PFU/mL, 5.27 × 10^5^ PFU/mL, and 3.00 × 10^5^ PFU/mL in cells upon p53 knockdown.

### Genome-wide transcriptional analysis of putative p53 target genes altered by Pifithrin-α in infected host cells

**FIGURE 3:**
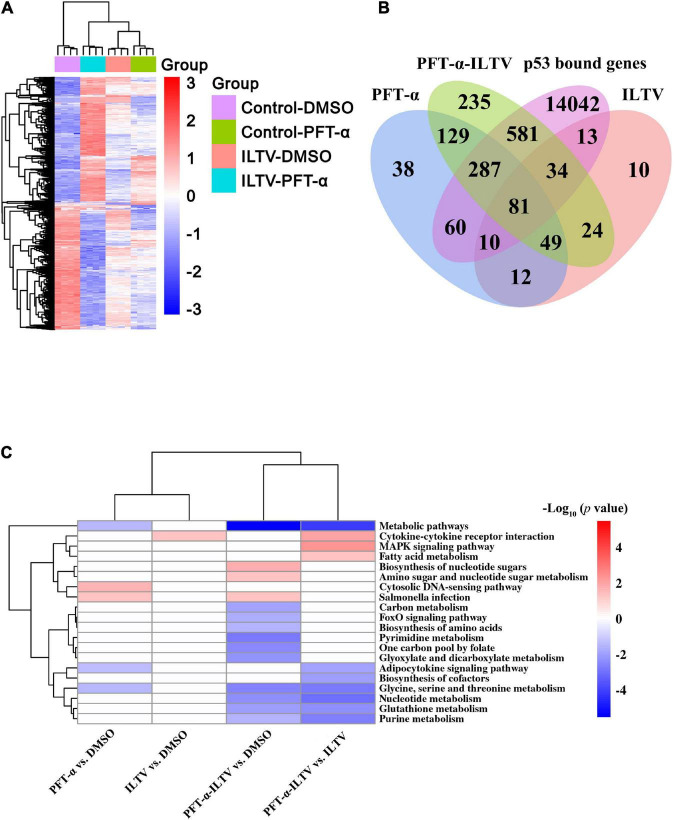
Genome-wide transcriptome analyses. **(A)** Hierarchical clustering analysis of 1,595 genes differentially expressed in LMH cells at *p*-value < 0.05, | log_2_fold change | > 1. Columns indicate arrays, and rows indicate genes. Values are normalized by row. Blue indicates repression, and red indicates promotion. **(B)** Bioinformatic analysis of the genes bound by p53 and differentially expressed in mock cells or ILTV-infected cells upon PFT-α or DMSO treatment. These DEGs of PFT-α treatment group, ILTV treatment group and ILTV-infected cells pretreated with PFT-α intersect with p53 bound genes, respectively. **(C)** Combined pathway analysis of significantly expressed genes (*p*-value < 0.05). Red indicates upregulated gene enrichment pathways, blue indicates downregulated gene enrichment pathways, and white represents unchanged pathways.

Next, we conducted the genome-wide transcription analysis to explore the molecular mechanism by which p53 inhibition decreased ILTV replication. Bioinformatics analyses using following criteria found 1,595 genes that were differently expressed between groups: (i) a *p*-value < 0.05, (ii) | log_2_ fold change| > 1. Using these differentially expressed genes (DEGs), hierarchical clustering analysis showed effective clustering of biological replicates. While, the transcription profiles of uninfected cells were clustered separately from those of other cells (Figure 3A). To address the potential biological processes directly regulated by p53 during ILTV infection, further transcriptional analysis with the genes bound by chicken p53 (Chen et al., 2022) was conducted. Among 15,108 p53 bound genes, 438 DEGs were regulated in PFT-α-treated cells, while 983 DEGs were regulated in ILTV-infected cells pretreated with PFT-α; 138 DEGs were regulated in the ILTV treatment group (Figure 3B). With a *p*-value of 0.05, pathway analysis using DAVID tools identified 20 pathways enriched by these DEGs (Figure 3C). PFT-α repressed 13 of the 20 pathways, and majority of these repressed pathways (8/13) were metabolic pathways such as nucleotide metabolism, pyrimidine metabolism, purine metabolism, and glutathione metabolism, demonstrating that repression of metabolic pathways plays a key part in the antiviral function of p53 inhibition.

### Identification of chicken p53 direct target genes involved to nucleotide metabolism and ATP synthesis

**FIGURE 4:**
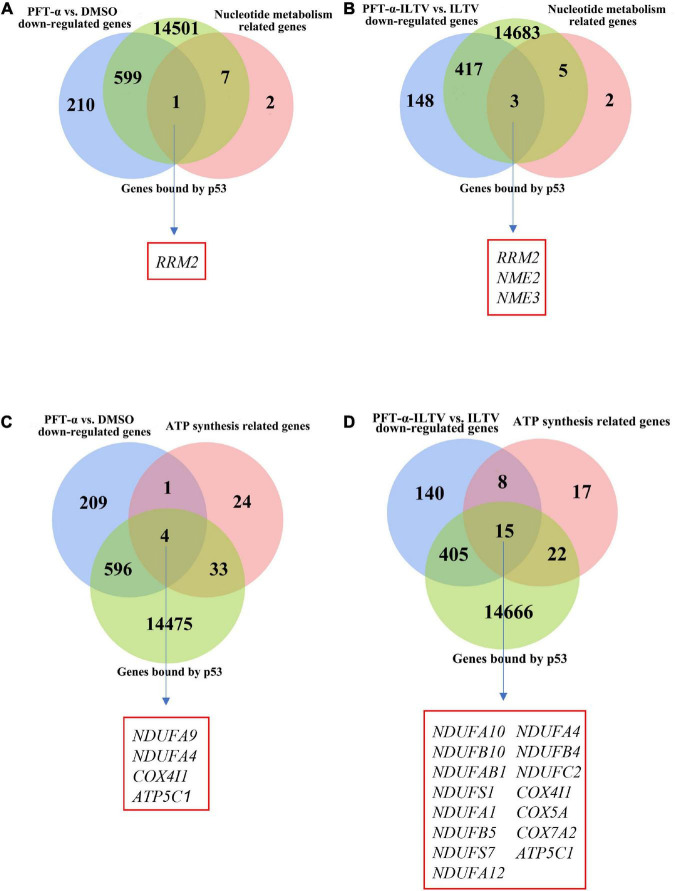
Relationship between genes bound by p53 and key nucleotide metabolizing or ATP synthesis related enzyme genes. **(A)** Venn diagram showing the intersections of these DEGs (*p*-value < 0.05) of the administration of PFT-α, nucleotide metabolism related genes and genes bound by p53. **(B)** Venn diagram showing the intersections of these DEGs (*p*-value < 0.05) of the administration of PFT-α in ILTV-infected cells, nucleotide metabolism related genes and genes bound by p53. **(C)** Venn diagram showing the intersections of these DEGs (*p*-value < 0.05) of the administration of PFT-α, ATP synthesis related genes and genes bound by p53. **(D)** Venn diagram showing the intersections of these DEGs (*p*-value < 0.05) of the administration of PFT-α in ILTV-infected cells, ATP synthesis related genes and genes bound by p53.

**FIGURE 5:**
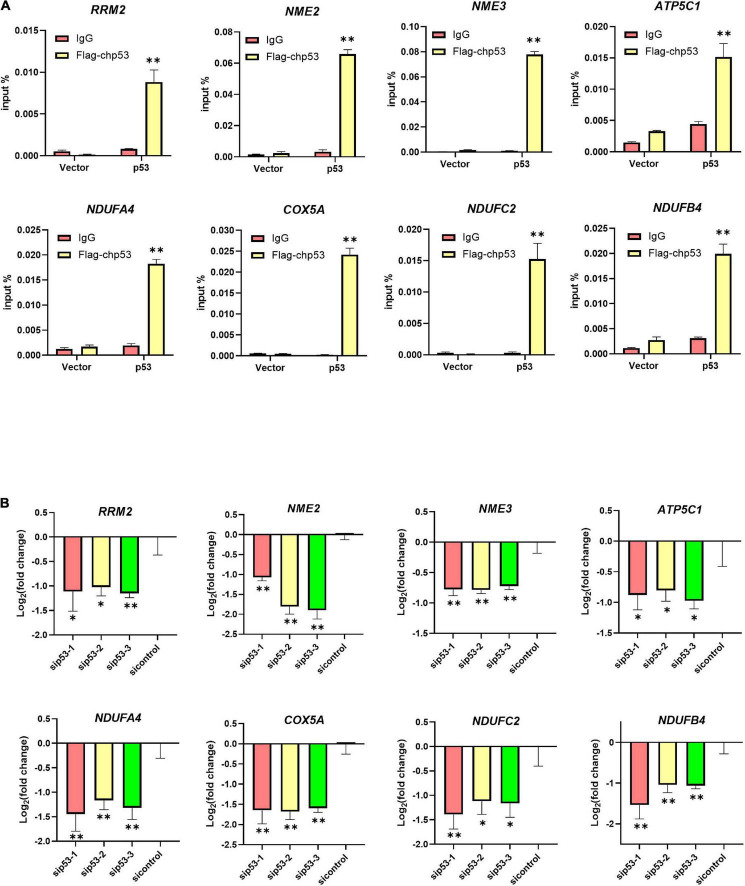
Identification bona fide direct target genes of p53. **(A)** LMH cells were transiently transfected with Flag-chp53 or empty vector p3xFLAG-CMV-7.1 plasmid (vector) and harvested at 24 h. ChIP-qPCR analysis of the relative p53 occupancy in the promoters of eight putative chicken p53 direct target genes including *RRM2*, *NME2*, *NME3, ATP5C1*, *NDUFA4*, *COX5A*, *NDUFC2*, and *NDUFB4* in LMH cells. **(B)** LMH cells were transiently transfected with p53 siRNA (sip53) or negative control siRNA (sicontrol) for 24 h and harvested. The transcriptional levels of eight putative chicken p53 direct target genes including three nucleotide metabolism related genes and five ATP synthesis related genes were detected by RT-qPCR in LMH cells. The results are presented as the mean ± SD, *n* = 3. *p < 0.05 and **p < 0.01 indicated the levels of significance.

Our previous metabolome and transcriptome analysis identified nucleotide metabolism and ATP synthesis as targets of PFT-α during its repression of ILTV replication in LMH cells (Xu et al., 2022). Ten genes related to nucleotide metabolism and 62 genes related to ATP synthesis were screened. As shown in Figures 4A,B, eight of the 10 nucleotide metabolism genes were bound by p53. Comparing with DMSO control group, the transcription of one gene, namely *RRM2*, among these eight p53 bound genes were repressed by the administration of PFT-α (Figure 4A). Comparing with ILTV-infected cells, there were three p53 bound genes, including *RRM2*, repressed by the administration of PFT-α in ILTV-infected cells (Figure 4B). The three genes are considered as the putative chicken p53 direct target genes. As shown in Figures 4C,D, 37 of the 62 ATP synthesis related genes were bound by p53. Comparing with DMSO control group, the transcription of four genes, namely *NDUFA9*, *NDUFA4*, *COX4I1*, and *ATP5C1*, among these 37 p53 bound genes were repressed by the administration of PFT-α (Figure 4C). Comparing with ILTV-infected cells, there were fifteen p53 bound genes, including *NDUFA4*, *COX4I1*, and *ATP5C1*, repressed by the administration of PFT-α in ILTV-infected cells (Figure 4D). The 15 genes are considered as the putative chicken p53 direct target genes. Among these putative chicken p53 direct target genes, three genes involved to nucleotide metabolism and five genes involved to ATP synthesis were selected randomly for validation. After p53 was expressed ectopically in LMH cells, the level of p53 bound to the putative binding sites was determined using chromatin immunoprecipitation followed by qPCR (ChIP-qPCR). The amount of non-specific antibody binding to DNA was measured using ChIP with isotype IgG2b antibody and input DNA was used as the negative control. In cells with ectopic overexpression of chicken p53, ChIP-qPCR revealed increased bindings of the overexpressed protein to the transcriptional regulatory areas of these eight genes (Figure 5A). While, p53 knockdown decreased the transcription of these eight genes as measured by RT-qPCR, suggesting the transcriptional control of these genes by p53 (Figure 5B).

## Discussion

Our previous study identified the specific inhibitor of p53 named PFT-α effectively suppressed ILTV infection (Xu et al., 2022). Here, we explored the effects of chicken p53 on each stage of ILTV replication and revealed the underlying mechanisms. We found that the repression of p53 transcriptional activity significantly decreased ILTV viral gene transcription, genome replication, and the formation of infectious virions. Chicken p53 seems to affect ILTV infection through its transcriptional control of host metabolism, since p53 target genes altered by p53 repression during ILTV infection were significantly enriched in the metabolic processes, especially nucleotide metabolism and ATP synthesis.

In fact, p53 can response to many different stress stimuli and regulates various biological processes, such as cell cycle, proliferation, apoptosis, epithelial-mesenchymal transition and invasion, pluripotency, ncRNA regulation, ROS control, inflammation, and autophagy, as well as antitumor and metabolism (Kastenhuber and Lowe, 2017). Till now, p53 has been confirmed as a key target in many viruses in both mammalian and chicken, such as Japanese encephalitis virus (Wang et al., 2020), hepatitis B virus (Song et al., 2021), and Porcine Epidemic Diarrhea Virus (Su et al., 2021); Marek’s disease virus (Deng et al., 2010), ALV-J (Yue et al., 2015), and IBDV (Ouyang et al., 2017). In this study, we found that p53 is a host determinant of viral gene transcription and subsequent viral replication of ILTV.

P53 regulates a wide variety of biological processes through its transcriptional control of its target genes. However, only a few genes, including *p21* and *MDM2*, have been confirmed as chicken p53’s direct target genes (Deng et al., 2010). Most chicken p53 target genes remain unknown, which impedes future study of the underlying mechanisms of chicken p53 functions. In our recent research, 752 novel chicken p53 direct target genes were identified (Chen et al., 2022). Using these chicken p53 target genes, we revealed host metabolic processes, especially nucleotide metabolism and ATP synthesis, both of which are essential metabolic requirements of ILTV replication (Qiao et al., 2020), suggesting chicken p53 as a promising target for the development of novel antiviral therapeutics against ILTV.

Our previous metabolomics study exploring the metabolic requirement of ILTV replication reveals that, ILTV needs ATP synthesis to provide energy and nucleotide biosynthesis to provide raw materials for DNA replication and RNA production (Qiao et al., 2020). In fact, this is common for herpesviruses, such as human HCMV and HSV-1, and evidenced by the fact that most clinical anti-herpesvirus drugs are nucleoside analogs, such as acyclovir, penciclovir, ganciclovir, and brivudine (Piret and Boivin, 2021). Therefore, the target genes related to ATP synthesis and nucleotide synthesis would be essential for ILTV replication. Considering the high basal metabolic level of this liver tumor cell line LMH and the multiple signaling pathways utilized by ILTV to regulate ATP synthesis and nucleotide synthesis (Qiao et al., 2020), further investigations with inducible knockdown of multiple p53 target genes involved in the same metabolic process in LMH cells are needed in the future.

Overall, our present study advances our knowledge of the interactions between ILTV and host cells, as well as the role of chicken p53 in viral infection, which will be helpful for the development of more innovative therapeutic strategies in the future.

## Data availability statement

The datasets presented in this study can be found in online repositories. The names of the repository/repositories and accession number(s) can be found in the article/Supplementary material.

## Author contributions

LX performed the majority of the experiments, participated in the statistical analysis, and involved in preparation of the manuscript. ZC, YZ, LC, ZL, and XL participated in the experimental work. HL and SL conceived the study, participated in its design, coordination, statistical analysis, and revised the manuscript. All authors have read and approved the final manuscript.
