# Supplementary material for: P53 maintains gallid alpha herpesvirus 1 replication by direct regulation of nucleotide metabolism and ATP synthesis through its target genes
Source: Front Microbiol. 2022 Nov 24;13:1044141. doi: 10.3389/fmicb.2022.1044141 (PMC9729838; doi:10.3389/fmicb.2022.1044141)
Supplement: Supplementary file 1 [file Data_Sheet_1.docx]

Table 1 List of RT-qPCR primers.

| Gene | Primer direction^a^ | Sequence (5′to 3′) |
| --- | --- | --- |
| *ICP4* | F | CAAGAAGTGGTGAGGAAGTC |
|  | R | AGGAGGAAGAGGAGGAAGA |
| *ICP27* | F | GGGCAGTGGATGATGATGATT |
|  | R | GATCCCGAAGACAAAAATGCTGG |
| *VP16* | F | TGAGGATGATGCGACTGAC |
|  | R | GCGAGAAACGATGCTGAAC |
| *gC* | F | AAATGCTACGACCTGAAACT |
|  | R | CTCGGGCTCATCCAAAACA |
| *gI* | F | CACTTTACAGCCGACAAAAACAG |
|  | R | CCAGGAACTTAGCGAGGAG |
| *gG* | F | GCAACCGCACCACGATTGAGG |
|  | R | TCCACTGCCCGTTTCGCTATCC |
| *P53* | F | ACCAAACGGCACAGCGTCGTC |
|  | R | CACACGCGCACCTCGAAGCAG |
| *P21* | F | CCCGTAGACCACGAGCAGAT |
|  | R | CGTCTCGGTCTCGAAGTTGA |
| *GADD45A* | F | CTTTCTGCTGCGAGAACGACA |
|  | R | ACCCACTGATCCATATAGCGACT |
| *MDM2* | F | ATTCTCAGCCATCTACGTCA |
|  | R | TGAGATGTCCTGTTTTGCCAT |
| *β-actin* | F | GTGGATCAGCAAGCAGGAGT |
|  | R | ATAAAGCCATGCCAATCTCGT |
| *RRM2* | F | CTCTCTGGAAGGCAAGACCAA |
|  | R | CTGTGGGCTTTGACATCACTCC |
| *NME2* | F | CGCACCTTCATCGCCATCAAGC |
|  | R | CCCTGCCTGTTTTCACCACGTT |
| *NME3* | F | ACCAACCCGGCTGAGTCCA |
|  | R | AGCTCCTCTGGGCGAAACCAC |
| *ATP5C1* | F | AGTCAGGAATATGGCAACTCT |
|  | R | GCTTTCTCATACAGTGCCAGT |
| *NDUFA4* | F | GCTCTGTACGTCATGCGTTT |
|  | R | AGCCAGTTTATTCCAAGGTTC |
| *COX5A* | F | ACGCCCGCTGCTACTCCCA |
|  | R | CGCTGGCAAAGTCATTTAACCGT |
| *NDUFC2* | F | TGTTCACCGCCAGATCCTGT |
|  | R | ATCAAGCAGCTCTCTGTCCAGT |
| *NDUFB4* | F | CTCTTCTGGATAGCCGCCTT |
|  | R | AAACACTGAATGGTCGCTCGT |

^a^ F, forward; R, reverse

Table 2 List of ChIP-qPCR primers.

| Gene | Primer direction^a^ | Sequence (5′to 3′) |
| --- | --- | --- |
| *RRM2* | F | GCCAGTAGTTTTCCACCTGT |
|  | R | TCCATATCGCTTCTGCCGTCA |
| *NME2* | F | GCTCTCTTTCCGCTGCCGCTCT |
|  | R | CCCACTGACTGCAGCGCACCC |
| *NME3* | F | AGTCTGAAACAAAAGCCAAGCTG |
|  | R | AACTACCACAAGCCAGTCTCG |
| *ATP5C1* | F | CCCCATCCCTGGAGGTGTT |
|  | R | CAGGGCCACCAACCTCCAC |
| *NDUFA4* | F | GCCCTCTCTTCCTCGCCGTTC |
|  | R | TACAGGCCCACAAATGGCAGA |
| *COX5A* | F | CAGCACCAACCCCGCCACA |
|  | R | GGGGACAAAGTTCTGCGCCGAG |
| *NDUFC2* | F | GCCGCCATGCCGTTCCTCCC |
|  | R | CGGTCAGCCCCAGCCACACC |
| *NDUFB4* | F | ACTTCCTTTTGCTTGGAGCCCTT |
|  | R | CGCGCTCTCTGCTGCCCTTG |

^a^ F, forward; R, reverse
